# Supplementary material for: Environmental and Socio-Demographic Influences on General Self-Efficacy in Norwegian Adolescents
Source: Behav Sci (Basel). 2025 Oct 31;15(11):1484. doi: 10.3390/bs15111484 (PMC12649750; doi:10.3390/bs15111484)
Supplement: Supplementary file 1 [file behavsci-15-01484-s001.zip › behavsci-3819387-supplementary.pdf]

Table S1. Hierarchical Regression Analysis for the Prediction of General Self-Efficacy Based on the Imputed Dataset (n= 21 580)

	Model 1 (R^2 = 0.202)			Model 2 (R^2 = 0.224)			Model 3 (R^2 = 0.229)		
	β	*B*	*95 % CI of B*	*β*	*B*	*95 % CI of B*	*β*	*B*	*95 % CI of B*
Parental involvement	-0.03	-0.02	[-0.03; -0.01]	0.00	0.00	[-0.01; 0.01]	0.06	0.05	[-0.02; 0.12]
Relation to peers	0.23	0.18	[0.17; 0.19]	0.22	0.17	[0.16; 0.18]	0.15	0.12	[0.04; 0.20]
Academic and social relation to teachers	0.15	0.12	[0.11; 0.13]	0.14	0.11	[0.10; 0.13]	0.04	0.04	[-0.04; 0.11]
Participation in physical activities	0.09	0.07	[0.06; 0.08]	0.09	0.07	[0.06; 0.09]	0.19	0.16	[0.08; 0.23]
Participation in music/cultural organized leisure activities	0.02	0.02	[0.01; 0.03]	0.03	0.03	[0.02; 0.04]	0.02	0.02	[-0.05; 0.08]
Perceived safety in the neighborhood	0.15	0.12	[0.11; 0.13]	0.11	0.08	[0.07; 0.10]	0.19	0.15	[0.08; 0.22]
Perceived access to arenas for physical and social activities in the neighborhood	-0.02	-0.02	[-0.03; -0.01]	-0.02	-0.02	[-0.03; -0.01]	0.01	0.01	[-0.06; 0.08]
Gender				-0.14	-0.16	[-0.18; -0.15]	-0.14	-0.16	[-0.18; -0.15]
Age				0.08	0.03	[0.03; 0.03]	0.08	0.03	[0.02; 0.03]
Socio-economic status				0.02	0.02	[0.00; 0.04]	0.02	0.03	[0.01; 0.04]
Gender x Parental involvement							-0.01	0.00	[-0.03; 0.02]
Gender x Relation to peers							0.07	0.03	[0.01; 0.06]
Gender x Academic and social relation to teachers							0.13	0.06	[0.04; 0.09]
Gender x Participation in physical activities							-0.04	-0.02	[-0.05; 0.00]
Gender x Participation in music/cultural organized leisure activities							0.01	0.00	[-0.02; 0.02]
Gender x Perceived safety in the neighborhood							-0.12	-0.06	[-0.08; -0.03]
Gender x Perceived access to arenas for physical and social acitivities in the neighborhood							-0.01	0.00	[-0.02; 0.02]
Age x Parental involvement							-0.11	-0.02	[-0.03; -0.01]
Age x Relation to peers							-0.05	-0.01	[-0.02; 0.00]
Age x Academic and social relation to teachers							-0.01	0.00	[-0.01; 0.01]
Age x Participation in physical activities							0.00	0.00	[-0.01; 0.01]
Age x Participation in music/cultural organized leisure activities							0.00	0.00	[-0.01; 0.01]
Age x Perceived safety in the neighborhood							0.03	0.01	[0.00; 0.01]
Age x Perceived access to arenas for physical and social acitivities in the neighborhood							0.02	0.00	[0.00; 0.01]
Socio-economic status x Parental involvement							0.05	0.02	[0.00; 0.04]

Socio-economic status x Relation to peers	0.06	0.02	[-0.01; 0.05]
Socio-economic status x Academic and social relation to teachers	-0.01	0.00	[-0.03; 0.02]
Socio-economic status x Participation in physical activities	-0.06	-0.02	[-0.05; 0.01]
Socio-economic status x Participation in music/cultural organized leisure activities	0.00	0.00	[-0.02; 0.03]
Socio-economic status x Perceived safety in the neighborhood	0.00	0.00	[-0.03; 0.03]
Socio-economic status x Perceived access to arenas for physical and social acitivities in the neighborhood	-0.05	-0.02	[-0.04; 0.01]

Note. Gender: Boys=1, Girls=2. Abbreviations: R^2 = Explained variance, β = Standardized Beta, B = Unstandardized coefficient, CI = Confidence Interval. Model 1 df = (7,21572); Model 2 df = (10,21569); Model 3 df = (31,21548).
